# Supplementary material for: Maternal Transcripts of Hox Genes Are Found in Oocytes of Platynereis dumerilii (Annelida, Nereididae)
Source: J Dev Biol. 2021 Sep 4;9(3):37. doi: 10.3390/jdb9030037 (PMC8482071; doi:10.3390/jdb9030037)
Supplement: Supplementary file 1 [file jdb-09-00037-s001.zip › jdb-1281402-SI.pdf]

Supplementary materials

Table S1. List of primers used in this study

Genes	Primers (5′→ 3′)	Size fragment (nt)
Pdum-Hox1 (3'- exon)	F: CCAGGAGAATTTACGTAC<br>R: GATGGAATGTTCACTGTT	270
Pdum-Hox1 (Intron-flanking)	F:GGGCACCATATGAACATGGG<br>R:GGCAGCAGCGATTTCTATCC	279
Pdum-Hox2 (3'- exon)	F: ACCAGCAGATCGACCAAAG<br>R: TCTGGGCATAATGTCCGTTA	208
Pdum-Hox2 (Intron-flanking)	F:CAAAGTCCCAGAATACGCCT<br>R:GAAGCAGCGATCTCGATCC	294
Pdum-Hox3 (5'- exon)	F: GACACTCATACAGCTGACAT<br>R: ATGAGGGTTGGCTCTCGTA	192
Pdum-Hox3 (Intron-flanking)	F:GGCCAATGGTCAGATGAACC<br>R:CTCTCACTCAAATTCAACAGTGC	447
Pdum-Hox4 (5'- exon)	F: GATTGGCTCACACTGACCAT<br>R: GCTGGGGATGTAATTGTTCTG	214
Pdum-Hox5 (5'- exon)	F: CTCATTACCGGGCCTATGG<br>R: GATCCAGCATGAGACAAGGG	355
Pdum-Hox5 (Intron-flanking)	F: AGCTGGTGGCAATTCAACTT<br>R: GTTTGTGTTCCTTCTTCCAT	392
	F1: TCAAGTAACAGTTCGAAC<br>R1: TCTCTCTGTTAAGTTGAG	222
Pdum-Lox5 (5'- exon)	F: TGGCCAGCTCTGAAGGAA<br>R: GACCTTGCAGCCCATTTC	269
Pdum-Lox5 (Intron-flanking)	F:CGGTCAGGCTATGGTATCAC<br>R:CGTGTGCGATCTCTATCCG	239
Pdum-Hox7 (5'- exon)	F: GGCACATTGGAAGATCATCA<br>R: CCACCTGGTCCTAATTGTG	181
Pdum-Hox7 (Intron-flanking)	F:ACGACAATTCCGTGGGCT<br>R:GTCAAACACAGCGAGTGAGC	229
Pdum-Lox4 (5'- exon)	F: CAGATATGACCAACAGTCTC<br>R: GAGATCCGATGGAATTTTCT	266
Pdum-Lox4 (Intron-flanking)	F:CCTTCTACCAATTGTTCTGGA<br>R:CCGTCAGACAGAGGGCGTG	253
Pdum-Lox2 (5'- exon)	F: GAGCTTTTATACAGAAGGAGT<br>R: GTAATGCATTGTACTGTCCT	227
Pdum-Lox2 (Intron-flanking)	F:AGATGAGTTCCTGTCTACCAC<br>R:TGAGACAGAGCATATGAGACAG	243
Pdum-Post2 (3'- exon)	F: GAGCAACAGTTACATCA<br>R: ATTTCTGGTGATGGTGGT	242
Pdum-Post2 (Intron-flanking)	F:GTACAACCCTAACCCAGCAG<br>R:CCACCGTTTGTTATCAATGTCC	414

Table S2. Probes size for in situ

Dig-RNA probes	Size (nt)
Pdum-Hox1	610
Pdum-Hox2	750
Pdum-Hox3	619
Pdum-Hox4	592
Pdum-Hox5	843
Pdum-Lox5	690
Pdum-Hox7	1440
Pdum-Lox4	740
Pdum-Lox2	720
Pdum-Post2	750

List S1. Sequences of *P. dumerilii* Hox genes. The color marks the positions of the primers from Table S1. The sequences of Dig-probes is marked in brick red types. The protein-coding part of the sequences is underlined.

Pdum-Hox1 (GenBank: AJ505022.1 + assembling of the 5' end)

GATTGGAGTTCTAGTAAATTATGAATACAAACGGAGAATATACAATTTGCAACTTGGATAATCACACA
TACACTTCGAATTATCCCACAGAGAATATTAGCACCACTGGATATACGTACAACAATGTGAATAACAC
TTTGGTGGACACTGGGCAGTTGAGTTATGGAGGAGGGGGTTACAACAGCACCATGGACAAGTGGAG
ATGCCCCATGGAGGGGTAGCCCACCACCCCCCTCACCTATACGGGGGTACCCACGGGGGGCACCATG
GACATAACAACGGGATCCTGAGCGCCGCTACCCCTCCCCCGACCCACCACTACATGCACCCGGCTGCT
ACCTGCCTCCCAAACTCCGGACACCCCTACCATGACTACTATAGCTCCGGCATGCTCAACGGGGGTGT
TGATGTGCCCAACCCTGCCTCCCCCTACCCTTACCCGGACCCCACTGCGGGCCATGGGCACCAACCCC
ACGGAATGCCCACGGCCGGGGGATACCCCCCTCACGGGCACCATATGAACATGGGCAACGGGGGCCA
GGACCAGGCCCCTGTAACAACCTATAAGTGGATGACTGTTAAAAGATCTACGGCTCAGAAAGGAACA
CAGGGTTATACGCAATCACCATGGGGAGGAAAAGTGAAACCAGGAGAATTTACGTACACACCGGGTC
AGCCCAATATGGGCCGGACGAATTTCACAAACAAGCAATTGACTGAACTGGAAAAAGAATTCCATTTT
AACAAATACCTGACGCGGGCACGCCGGATAGAAATCGCTGCTGCCTTAGGACTGAATGAAACGCAGG
TGAAAATCTGGTTTCAAAATCGGCGGATGAAACAGAAAAAACGAATGAAAGAAACAAACGTGAGTCC
CACAACGAACGGTTCCACAGAGAACAGTGAACATTCCATCGCCAGTGACGATAACAGTTGACCGGTG
ACGCCATGAAGAGTCTT

Pdum-Hox2 (GenBank: JQ424893.1)

GAATGTCTCAAAGGCTGACAGGCCTGTAGTGCCAGAGACAAGTTTTGACAGCAGATAACAGTACTAC
ATATTTGGATACATATTATAGATAAACATTATTTTAAACAAACACTCAATGGACTAACAACTTATGGCT
GTGGACTTCAGTTATAATCGGACTTACAAACTTTTCATGCAATACAGTGCCTGTCAAAAGTTTTGAAGA
AACTTGGACTTATTTTTACACATACTTTGGAGATTTTGGATCGACTTATTAATACTTTCAAATACAGTG
GATGTCATTGACACCCATGCAGTTTAACACAACTGTTGTGTCGAATGACAATTAGATGCACAACTGAC
TTCGGACTAATTTCACAAGGGTGTCCACATGGTTACATGCCCCGGTACAGGGCCCACGGCCCATTTGG
GGCCCAGGCTACCCCCCTCCTGCTTGCCTGACCCCTCTTTCCCCGAATTCATGATGAATATACCACACA
TCTCGGAGACTCTACACCACCAACAAAACTCTTTACAGGGGACTTATTACTTACACGAAAAACACTTG
TCAGCAGGCCCACCCAGCCCCAAAGTCCCAGAATACGCCTGGATGAAAGAAAAGAAGGCTGTCCGCA
AAAGTAGCCCCGGTCTTCTGGCCCCCCATCCCCCCGGTCAGACTATGGTGGCTTCTCCCGGGTCTTCTC
TGCCATCACATCAACCTGACTTTGGAGATGGCGGAAGTGATGGAGGGAGTACCGGAACAGGAAGCAA
CCCTCGTCGACTACGCACAGCTTACACAAATACCCAACTTCTTGAACTGGAGAAGGAATTTCACTTCA
ACAAGTACCTCTGTCGACCAAGAAGGATCGAGATCGCTGCTTCACTGGATCTCACTGAGAGGCAAGTC
AAAGTCTGGTTCCAAAACCGCAGGATGAAGTTCAAGAGGCAGACCCAGACGAAAGGCGTCGGAGACG
GCTCTTCCGATGACAAAGTGACTTCATCTAGTCCTGCAATCGACAGCACGAGCTCTGAAGATGGTGAG
GCAATGAAAGAAAGTAAATCAGAGTTGTCTTCAGATGTTGAATCACGGTTAGGTTCTCCTTCTGAACT
CTTCATGAAGAAAGATGAGAAGGAAGATGTCGGTAGCATCGAATCAGTTGGAAAAGAAAACCGTCAT
CCAACAGATACCGGAAGTATCTTATCTGTTCCAGAAGATCAGAAGTCCACACTTGATCTTGATGATGA
TGTCTTGCTCATCAAAACGACTGATAAAGAAACGAGGTTACCGACAAAAGAACGACCTGCTAAAAGA
CGGTGCATCCGCAAAGATTCTTCAGCAGTTTCCCCGATGCCGAGTCTAGCACCAGAAGCCTTCTCAGC
TGGTATGACAAGGCTCTCAAGTCCTTTGCCACCACAATCATCCATGCCTAATATGTGGGGTGGTTATTC
CGCTAACCAGTGCTCAGACTTGTCTCAAGACTCTTTCGGTTTCCAAGGCCATCGCTCGGCCCACCAGCA
GATCGACCAAAGGGTACCCCATTTCTGGTTCACACGGACTCTCCAGACAAGGGAGTTATGGTTACGGT
GCATCTGGAATGAACATGTCTAGAGATGTGGATATTTCTGGATCCAGGAGTCCTGGTGTTGTTCAAAA
TAACATGCCGGATCATAGATACAATCCGAATGGACAACAAGGTTATAACGGACATTATGCCCAGATG
AGACAGGAGATGAACAACTTCTACCCGCCCCAAGCTTACAGAACCAGAATTATCCCCCGACCCAGATG
CAACATTACTCAGATCTACCATGTGTATACAAACTCTAGTCAGCTCCTCAGTATACTATACTATAGCAA
GGACCTATGTTCACCCCGAACAACATCCGACTTACCGATGACCTAAGGACCAAGTCAATCGGACCGAT
CCTTATCGAACGTGCGAATGCAGTTCTAA

Pdum-Hox3 (GenBank: JQ424894.1)

GACTTGGTCATTAATAACCTCGCTGACTACCCGCTCGGGTCGATGTCTCGAAGTCTCCCCGGGGCTCTT
GGTTTTTCATCGCTATTTATCGAAATTATATAGGTTTATACACACATATAATTTAGACATTGGACATAT
ACCTCAAGCAATTAGAGCTTGATCATAATTAAGCTATTGGACTTTACACATTCGGAATGATTTATGCAA
ACTTCAAAACTTCTGTGCGTCTTTGAAATCAAAACTTGGATTGAATCTTTGGAGTCAATTGGAAAACAC
TTCTGACAAGATTGATGATAATTTCAGCAACACAGTATCTGACTCTAGTTAATGGATTCATAACTATTG
AAAACTATGAATCTGTGAAGTCTTTCGTTGGAAAACGTTGCCAATATACCAAAAACATAATACCATAG
TTTTGACACAATATACATTGGTTCCAACACTTCAAACTTATTGGACACAATTAAAGATTGCATCAATGA
TTGGGAGCATCATCGCCAAAATGGAAGTCAACGCGAGATAGAAACTGATTAACAATTTCACGCCATTA
TTCCACCATTTGAGACACATCAAGCCAAAAGTGAGGCTCAGGCCATCCCAGAGTGAGGCTGGGCAATC

CAAAGTGAGGCTGTAAAATCCAAAAGTGAGGCCTGACCATCCAAAGTTAAGGAAGGACAGAAGCCAG
GAGATTAAAGAGCCATTCTTCAAGGTCCGGGTTCACTGTGTGACTTCCAGAGAGTAGTGTGACATCAT
AGAAGTGACCTCTCAAGAGAGATTCCTAGAGTCGGAACAGAAGACATGGAACCAGAGATGAGTTGGG
AATGACAGTGAACACACCAGCTCAAGACATCACCATTGGACTATATACCTTCTCTGACAAGCCTCTGT
CATCTATACATGTATGAGGCTGCAACTTTAAGACACTCATACAGCTGACATAACTTCATATGTACCTAG
ATTTGCTAAATATCTGATTGACAGTTGAAAGTTAAGTCAGTCACAATGTCAGCACCAATCAATGACGT
CCCCTACAGTGCTGTCAACTCCGTCACGAGGGATCCTATGCACCTTCAGAACCCCAAAGGACCTTATT
ACGAGAGCCAACCCTCATTCCATCCTGGATTTTACCAACCCACAAATGGCTATCCGGGATATATGGGA
AGCTATGGAGGGGATCAGCCCTCCCTGGATCCTTACAGACCCCAATACATGGCCAATGGTCAGATGAA
CCAACATTGTTCACCCCCTATGGACTACAACACATTCAACCCCTGTATGCAGTATGGGATCCCTGGTGA
CTCCATGGGGCCGGGGTTGACCCCTGGAGGACCCCTGATGATGAAGGGCCCCCCTTCTGGCCCCTCGG
AAGTCTACCCCTGGATGAGGGAATCCCGGAACAGCAACAAGAGACCCAACCCAGGGAACACCTCAGG
ATCAGATGATGGAATTAGTCCGGAGAGCGCCGGACCAGGAACCGGAAGTGGAAGCATCAACGGAGG
AGACTCGACGAGCGGAGAGAGCGAGAAACCGAGCAAAAGAGCAAGAACCGCTTATACGTCAGCCCA
ATTGGTGGAGTTGGAGAAGGAGTTCCATTTCAACAGATATCTGTGTCGGCCGAGGAGAATAGAGATGG
CAGCACTGTTGAATTTGAGTGAGAGACAGATTAAGATTTGGTTCCAGAATAGACGCATGAAGTACAAG
AAAGATTCGAGGTTGAAGCCCAATTCTGAAAAAGAGATGAGCGAAGATGGGGAATACGGAGGAAGG
AGTATGGAAGATCTCTTTCCGAGTGAGAAGGGTGTCCCCCTAGGTCATTCTGGACCGATGTCCGGCCC
TTGCGAAACTCCTCCTTCCGCCAATACACAGATGCCTCCGAACATGCATCAGGAGCAACATATGCAGC
AGCACCATAATCAACAACATCCTCACAGTCAGCATACCCAACCTCATCCGCATAGTCAAGCTCACCAG
CAACAGCATTTACAACAGCCAATGCCTCATCACCCACACAACAACCCGCAACAACATTTACGACAGCA
ACATCCTCACATCCAAC

Pdum-Hox4 (GenBank: JQ424895.1)

GGCCAGCAGACACGGAGCCTTTTAGATGATTCATGGCCACAGGACGAACAGGGTGGAGGGATCTTCC
AGAATTTTAGGAATCCAGTTTAAAGCTTTCTAGGAACAGCTTTGCAAGCAGACGACGCGAACAGCTGA
TCAAGTAAACAGCTACACTACAGAGCTGCAGTGATGGTGTAGTGCGCACTGTGAAATCTACTTCTTTT
ACAAAAGGATTTTGTGGTTTATAAGTAAAAGACAACTGGAGAAAAACAATTGTGCAACTCTGGACTAA
AAGACTTGTCATTTAGATCTTCAAATCCATACCAGGGATTATCAAGTTCAGTGGAGAGAGCATACAGT
AGTTTAACTTCAACAACAAACTGTCAGTCTTCTAACCTGGGTCAGAGGTTATAGTGGGGCCCTTTTGAT
TGGCTCACACTGACCATGTGATATGTAAATAGCAACATACAGGGAGGGTCTATCTGTGCGTTGGACGT
CAGTGTGTGAGAAAAATTATCACTACAGAGGGATATATACTATTAATCACAAATGAGTTCCTTCATG
ATGAACTCGAACCCTTACGCGGAGCCTAAGTTCCCACCAACAGAAGAGTACAGTCAGAACAATTACAT
CCCCAGCGGACATCCGGAAGAATACTACAGGAGTCCTACAGGTTACGGGCCTTACGATGTCCGGAGGT
ACCAGGACGGAGGGGGATACCCACAAGGGCCTGTGCACAATATTTCRCCKTATGGACAGAACCCAGG
AATGGTCACGGGGACGGGCCCCCCTCCTGCCCATATGCCCATGGAAAATCAGAACCCAGCTCATCAGA
ACAGTTACTCTAGCCCTCCTCCCCCTCCCCACTCGGACAGCCCCGGCAGTGTCCCTTCCCCGACCCCCC
CTTCATCGAACCCCGGAGTCAACAACTCACAGTGTTCTCAACCTAATTCCACCCCTACAAACCCTCCTG
TTATTTACCCCTGGATGAAGCGGATACACGTCGGATCACAAGGGGCCAATGGAGCTTACGGAGCAGA
CAACAAGCGAACGCGGACGGCGTACACGAGACACCAAGTCTTGGAACTGGAGAAAGAATTTCACTTC
AACAGATACCTGACCAGAAGGAGGCGGATAGAGATCGCTCATGCCCTCTGTTTGACGAAACGGCAAA
TAAAAATCTGGTTCCAGAATCGCAGAATGAAATGGAAGAAAGAGAACAAATTGCCAAACACGAAAAA
TAGACTCAGTGGCTCCTCGGCCAACAGCATCAACGGAGAAGGGTTAGGGTCATCCGCTCAGGACCTCA
GCCTCTCTCCGCCTCAGGGAATGACCTCACACACAGAACTCCCTGACGACCTATCGCCCTGATAGTGA
GTCCTGACGTCATGAAACTCATGATTCCTGACGTCATGGTGGCGTCGTTGTGTGATGTCAGCACTTTGT
GACGTCACAAAACTGTTCGTTATGATGTCATCAAATGCAGCAGCAAATCGCAGCCTTGTGGGATGCTT
ATACATGACGTAATCTTGGCGTAAACAATAACAATAGCCTCAGACAATATGGTGACAATATATTGCAA
TTCTTCGATATTTGTTTTGAAGTTGAAACTCTATACAGAAAATCCCAAAGCAAATGTATCCCAAACATC
AACATGGTTCCCTCACTGGTTCCAATGTTTTGGTTATCTCACAACCAAATTGGTTCCCTTATTCGGATCC
GATCTTTGGACACCGTGATGTTCCAGTGAACCAATTCTGGTTTCGACATCTGATCCAGATTTGAACACC
AAAAAAGTGATTCAGTTGTCTTAATCATTGTTCGAAACGGGGGAAAAGATGGCAGTTTGCGTATAGAT
CCCACCACATTATTATATAGGCCTATTTTTGTAATAAGGCTAACTACATAGAACATCTCCATTCTCTAG
TGACCTATTCTTGCCTTGAGAAAGAAGATTATTACGACTAAAATAAAAAAGTACTCTGCGTGATACCA
CTGCTTAATCGAATTCCGCGGCCGCCATGCGCGGAGCATGCGACGTCGGCCCATCGCCCTATAGTGGA
GTCGTATACATCACTGACGTCGGTTTTACACGTCGTGAACTGGCAAACTGCCGTACCACTTATCGCCTG
CAGCATCCCCTTGCCAGCTGCCTAATAGCAAAAGGCTGACGATGCCCTTCACAGATGTGGCCA

Pdum-Hox5 (GenBank: KY020043.1 + assembling of the 5' end)

TCATCATCCGTGGATCTTTATTCTGGGGAAGTTTGAGGGGTTACGAGCCCACAGGAAGGACTTATGAG
TTCGTACTTCGTCAATTCACTGACGACATGCTATCGTCAGGGCGTGGGGGACGGGGGAGGAGGCACAG
AGAGGGGACCGCGCGACTACCCCCAGTCGGCCAGTCCTCATTACCGGGCCTATGGAAACGCTGGATAC
TCGTACGGATCCCCTGCTGGATCTAACAACCCCCCTCAAAATGGCGATTATTACATGACACAGAGACT
GAGCCATCCTCCACTGAGGGACACGACCAGTCCAGCGGCTTCTCTTGCAGTTGACACTGGCTCTGGGC
CCAACGCTCTGAGCGCAGGATCAGGTGTGAGGGACTATTCCAGCAGTTCGTACGGGAACAGCAGCAG
TGGTGAGCTTAGTCCGATAGATTCACCTCCCCCGGCTCACCAGCACCATTCAACAACATCACACAGCA
ACAGCAACAACAACAACAGTTCAAGTCACCACCCCTTGTCTCATGCTGGATCCCACGGCCCATCAACA
AACTCCAACAACAACAGTTCATCAAACTCCAATAATAACAACACAGCAACTACAAATATCAAGTCGA

GTCCTGGAGCAGGCCCCCCTCAAACAAACAGCCCGGCAGCTGTAGGAGCTGGTGGCAATTCAACTTCC
CAGACAACCGTACCTTCCCCCGACAGTTCAGCCAGTCCCGGGGCCAGCTCAAGCCAAGCCAGCCCAGT
TGGATCACCAGTCAACAACAATGGCTCAAGTAACAGTTCGAACCCACAATCTCAACAGCCACAGATCT
ACCCCTGGATGAGGCGGATGCATACTGGTCATGAAGCCAATGGGGTGGAGTCGAAACGGACGCGGAC
GTCGTACACGAGGCACCAGACTTTGGAGCTTGAGAAAGAGTTTCACTTCAACAGGTACCTGACCAGAA
GGAGGCGGATAGAGATCGCCCACGCGCTCAACTTAACAGAGAGACAAATAAAAATCTGGTTCCAGAA
TCGCAGAATGAAATGGAAGAAGGAACACAAACTGGCTCACTTAGCCAAGTCCCAGGGAACCAAGTTG
GACCCAACCGGGGGACCGTTAGACATGGACCGGAAATCGTGAAACATGACGTCACAATGGTGGTCAC
CTTACGAAACTGCCCCCCAGCAGATGCCGTCTAACAAACCGTTAGACTATGTCTAATGACTGACCTCA

Pdum-Lox5 (GenBank: JQ424896.1)

ATGGCCATGGTTAGAAGAGTTGGTGAGCCCACAAATCAAACTAGGCCTGCAGAGATTTTTACAGGAA
GGACAATGAGTACGTACTACGGCAATATTCTGCCGACAAACTTGTCGAATGGCACTAGCCAGGAACAT
TACATGGCCAGCTCTGAAGGAAAGTTTGATGCCTCCTACTTTTCTGGGCAAGGTATGGGGTTCGAGGC
AGCTGCAGCGGCGGCAGCGGGGGACGGCCAGCACCCTCACTACCCGAGGTTCCCCCCGTATGATCGA
CTGGACATCAGACCTATAACTTCAATGGGAAAGGGGGCATATACCCCTTCTCCTGCACACTACCAGGC
TTCTGGACTGAACAATTACCAATCTCACCACAACGGACAATATTCTCCGGAAGAAATGGGCTGCAAGG
TCCCCCCTGATGGGATGCCCAGCCCACACCATGCAAGTCCAACCGGTCAGGCTATGGTATCACCTTTT
GCCCCGAACAATATGCCTGGCTTGGTGAATGGTCAGCAGGCCCAAAACATCCCTATATACCCTTGGAT
GAGACCTATGAGCGGAGTTGCTGAATTTGGTTTCGAACAGAAGAGAACAAGGCAAACGTACACACGC
TACCAGACTCTCGAGTTGGAGAAAGAGTTCCACTACAACAGATATCTAACTCGGCGACGGCGGATAG
AGATCGCACACGCTCTCGGCCTCACAGAGAGACAGATCAAGATCTGGTTCCAGAATCGCAGAATGAA
ATGGAAGAAAGAGAACAATCTCGCAAAATTGACGGGGCCAAACGGCGAGCCCAAACCTGCCCTTCCC
CGAGTCGACGACAAAACCCCCGCCGACTCCTCATCACCCTCCCTCGCGACAGACCTCTCACTGTCAGG
TACCAGTCCCCACACAGACAATTCAGACCCCTCATCTCCCCTCGGCAGCTCTCCTTCATTATCGAGCCC
AGAAGGAAAAATGGGATGAAATTTTCAGACCGGAAAGACTAACTTTCGATCCCACT

Pdum-Hox7 (GenBank: KY020044.1)

TCGTTGGGCAGAGTTGGCTAACTCCCTACTCCCGTGCGAAAATAGTTCCGGCGGTTTGGCAAAAAAAT
CCAACATGATACCGCTTAAAACTTAAAAAACTGCAGCCCACAAGATTTCCTCTTGTTTGAGCATAGTTC
GTATTAAATTTGACCATATCTCTGTGTGTTTTGAAGGAGCCGAGAGCCGAGAGAAGTCGGGGACAATT
TCTGCCAACAATCGGGACTCCAGGCCAGCACGACAAGACAGATATACATCAGGGAAAAAGAGAAGAA
AAAAAGGAAAAGGGCTGACAGAAAACTGCCAAAGAATTGGAGTGACCTAAGTTTACCATCTTGAGAA
ATTCGAAAGTTTGGCAAAGAAGCTGAAATTGAAATTCTGAAAATCACACAAAATTTGGACCAAAAGG
TCACCATGGTAACCAACCTAAAAGAAGTGTGAAAAGACAAGAAGAGGAGAAAATCAACTGCAAAAA
CATTGAAAAAAATCGAGAAAAAAGTAGGAGACAAGAACTTCAGATCATCCCTGTATTACCAGGGACC
GAATCGATCAGGGCACATTGGAAGATCATCATAACTGGCCCTTTTGGCCAAAAACTTCATAAAAAAAA
AGACTTTTTCAATAATCAGGATCGAAAATTAACAGTTCANGATGAGTACATATTATACTAATTCCTACC
TGACGGGATCAGGTACCGGATCAGCTAGCTATGGCTCACAATTAGGACCAGGTGGAGCAGCCTTCGG
GGGCACGGCAGGAGACAATCGGCGGCCCCAGGACGGATCTCCCCTGTACGCCGGGCCCAGGAGCCCC
CCTGCTACCAGTGAATATGCTAAATATGGGTCGTATAACGGGCATTTGGGGTCCCCGACGAGCCCTGG
GCCTACGAGCCCTGCTTCCAGTACGAGTGGAAGTCTTTACGGAGCTTCAGGGGCAGATTATCGACGAA
TCCAGTCTCCTCAAAACTCTGGGTTCAGTCCAGGAAGTGGGACTGGTGATGGCCGGCTACCCACCAGC
TCGCCGACGCCCGGGCAGCATCACCGCAAAGACCTGGGCCCCCCTTCCCCCGTGGCTCACCAACAGCA
GCTTGCACCTATCAATATGTCGAACCACTCGAAGCCCAAAACGCCGACACTCGTAGACCGCCCTGCTT
CTACCAGCTCCAGCACTTCCCCTCCCGCTGGCGACGATCGGGATGAGGGGGAATTCGACGACGACAAT
TCCGTGGGCTCTTCCGGTGAGAACTCCAACACCGGAAACGGAAGTTCATCCTCGAACATCCCCATCTA
CCCATGGATGAAGTCCCAATTTGGACCAGAGAGAAAACGTGGGCGCCAAACATACACACGCTACCAA
ACTTTGGAACTCGAGAAAGAATTTCACTTCAACAGGTACCTGACCAGAAGGAGGCGGATAGAGATCG
CTCACTCGCTGTGTTTGACG

Pdum-Lox4 (assembling de novo)

GCCACCAGGAGGGGCTTCGTCAAGTGAAAAACAGCTCGCTGTATCTTGCCACCAGAGTGGAGGAGAG
CTGGACCACAGGTATCTGCCATCCATATATGTCAGAAGAGACCACGTGATTCAAGATATCATAATTAA
GAGGCCGCTCGCAAGCTTGGAATCTAATTAGTTTTTTTGGCGCGCTAGCTTATATTCAATGGTGAAGGC
AAGTGTTTTGCTTTTCCCCACCGTGCAGCGCTACCCTGGCCACTTGGATTGTAAGGAGGGAAGGAGAT
AGCTCTCACACTGTGTTTCAGCCTCACACACACWSTGTTCTGCTTCGCTCACACAAGCCAGCTCCCATA
GCCTCCTGGACGGAGCCTTCTGATTGGCTAGTGCAGCTGACATCACATTAAAGCAGAGGGATCTGGGT
AACTGAACACATTGCACAGACCAGTAACAAATCATTTTGTGTTCCCGTATATACCAGTAGTCCTATAG
CATTCAGCAGCTGAGATGAGTAGCTGTAGCTGCTGTTAAAAAATGAACTCATATTTTCCTAACCCCTTT
CTCCTCCAAGGAGACGGACAGTCAGCAGGAGGAGGTGGTCTTCAGTGCAATTTTACAGGAATATCTAG
CAGATATGACCAACAGTCTCAGGGGCTCTTCTGCTCCAACCCCGCTATGTCGGGCCAGTATGGATCCA
CAGGAAGCCCAACGGGGCATCCCGGACATATGGGAGACAGCAACTGCAGACCAGGAGATCTGAATGG
GTACGAAAACAGCCACCACCACGTGAACGCGGGCTGGTCAACCCCGGCTGCTGCAGGGGCACAACAA
CCGGACTTCAGCAGCCACGGGGGCCCTTCTACCAATTGTTCTGGAGAAAATTCCATCGGATCTCCAGT
AAACCACAACTCGTACAGCAGTCCAGCCCAACAGACAGTCCCTTTTTACCCCTGGATGGGTGTCGTAG

GACCAAATTCCTCACAGCGAAGAAGAGGTAGACAGACATACAGTCGATACCAGACGCTAGAATTGGA
AAAGGAATTTCAGTTCAACCATTACTTAACTCGAAAACGGCGGATAGAGATCGCCCACGCCCTCTGTC
TGACGGAACGCCAGATAAAGATCTGGTTCCAAAACCGGCGCATGAAATTAAAGAAGGAGCGCCAGCA
AATCAAAGAACTGAACGGGGAAACCATTACCCGAACTTCGCTCGGCTCCAAGTCGAATGCTGACGATT
GCAGCGACAAGGAGCTCGACGACGAGTCGCCGTTAGACAGTCCCATAAAAGACTGAACGACTATGGA
CAGAATTATATATTATAGGAGTTGTCAGGGCTATAGCATGGTTGTGCACTTGGACCGATTTGAAATAC
CAAAAGCGTCAAACAGCGTCACACGGCTGTGGTTGATATTAATTAATTATTAATCGCTGACAGCGCGG
TGGTGCTCGTTGGGCACAAAAGGACAGCGACCCTTTTATGAGGGTTAAACTAAATGCCCCGTTGTTGT
CACAGAGCAACRGCACTTCTACGATGACGGCGACACCAWAACAGTGCCGACAGMAACGTCAAACWA
CGTCAAANACAACGTAAAACTGCAGTGCAACAGCGGGGCGAAATATTCAAAAGACTTTGATGTCGTA
TTTAGTCAGGTAATACAGTGCTACTCATCGTGGTTTCTCGAGTTATGGATCTGTGAATTTCCAATCACA
CCATTGATATTACTAAGGCATTCGACAAGGTTGTGTTGTTTCACCAACTCCTTTGTCAAATGCTTCTTTG
TGGTCTATTCTCACAATGACTCTTCACTTCGAGCAGCAAGCTGTGTCTACAAATGAGAACTCTCCACAT
CTGGGCAGAACCTGCATGGGTTTAATAATGATTACCTATTGATACTCA

Pdum-Lox2 (assembling de novo)

CGGGTCAGACRGGCCGATGTGAACAAGTAGCCAATAGTTTGAATAGAGCATGTTGGGAACAACCTCA
AAGCTACATGCACGTTTATAATGGCTAGAATTTGGTACTAAAACAGCTGTTTACATGCCATACACTTGG
ATTATTTTGTGAGTGGATTTTTCCTTCGAAATGAGCTTTTATACAGAAGGAGTAAGCGCAGCTACTGAG
GATGTAGTCAGTTCCAGGAGGCCGACGAGTGTTCAAGCTGGCAGGACTGAAAACACCTCTCTGTCAAT
AGGGGCCGCCTGTGAGGCAGTCAATGGAAACTCTAGCCCGCGAGTTCCCGTCAGCTTTAGCTCCCAAG
TTTATCAGGACTACTGTGGATTAACGGGCTACCAAGGACAGTACAATGCATTACATAATAACCAGCAA
CAGCAATCAGGTAACAGCCATGGAAACGGGGGTAATTACCCTTATTTAACAACAGACAATGGCCACC
CCAACCTCCATGGTATGTCCCCGGTCGCACTGGCTCTTCTGGTTCCACAGGACAAAGTCTTGATGCAG
ATTTTTTGGAGATGAGTTCCTGTCTACCACAACATGCTGGCCTAGCTGGTAATCCAACTAGAATTTCTT
CAGCGCCCAGTCTCCCACCTATGTACCCATGGATGGCCATAGTCGGTCCAAACTCAAACCAGCGACGC
CGGGGACGCCAAACGTACACTCGCTACCAAACTTTGGAACTTGAGAAAGAGTTCAAATTTAACCGCTA
TTTAACTCGAAAAAGGCGCATAGAACTGTCTCATATGCTCTGTCTCACTGAAAGACAGATAAAAATAT
GGTTTCAAAACCGGCGAATGAAAGAAAAGAAAGAAATTCAGGCTATTAAAGACCTAAACGCYGAAAG
AGAACCATCCACTACGACCCCCTCGACTGAAATCAAAGATTAGCCTCACTAGGGGCGCTGCAGTGCCC
CAACATGGTGTCTGGCGGGCTTTTTGAATTTTGAATTTGATGTAAACAGTGAAATGACAACACAGTGA
TGTCAGTGTAACTCTCTACATTGGACTGTGTAGATAAGTTAAATGTTTTTGAAGAAATTGAATCAATTC
TATGATGGTTGTTGGCATTTGGGTGTGCAAGATGTGATGCCATATGCACCTGAATTAAACTGTTCTTGT
GCAATGGTTAGGCAATATTAGGTAAGTTGTGTTTGAAGGGCAGTGGTGGAAAATATGGTTTGTATATC
AATGACTTTCTCGCAACCTCTGGTTGGTAATCAATGTGGTTAGCAACTCTGGTTAGCAACCTGGTTAGT
AACCAGCTAATCAGGTTGGTAATCTAGTTGGTAATCTGACATGTGCTACATTATTGCTGTTGTCATGGT
ATGTGCCAGTGGATAATTATTGAAAAAAATTGTGACATAACAAATAGTATATCATATATATATTATGT
ATTATCTATGGTGATAAGTAAATAACAGACAACAGCTACTCTCTCAAAAACAAAGAGTCTCAGAGATT
ATGTAACTTTATATACGTTACTAACATTGTAAGTCATATAACTTAAATTTTTTGTCAAATCAGTGAAAA
CCTACTACTGCTCAGCCAATCATGGTGGAGCAAAGAGACTGAGCTGCTATTTGTAGCAAGTGTTCATG
ACGTCATAGACATTTTGATGACAAAGCAAAGCAGCATTCTTTTCTATGTGTTCTGATGCCACAG

Pdum-Post2 (GenBank: JQ424898.1 + assembling of the 3' end)

GACACACTCTGCTTCATGTACGGAAAAGCAGGTATCTACAGAATTTCCCTGGAAAGACTTCAGAGTGG
CACAAGTGCTGCAATGGAAGGGCCGGCGAATAACCTTTTGGATTTAGCTCGGAGTGCTGCTATCAGCC
CCTACCAACCCACTACAGTCTCAACAATGGCTAACATAGTGTCGGCCAACATCAGTCCTCAGGACACT
AGGACTCACAGGCCCCCCTTGAATGGGTGGGCATCTTACCAAAACGAAGTGAGTGCAGCAGCCGCAG
CAGGGTGCAATTTCCCCCCTTATGCTCCCCACATGGCCTGGGAGGGGGAGGGGGGAGCAGCCTCCCCA
ACAAACCATCCCCTTACCCTTCTTTTACCACTGGATCAGACTTTTTGCCAAATTGCCAGCAAGTACAAC
TCAACCAACTCAGTCCACTCAACAGCTTACCTCCCAGGAACTTCTCTTTCTATGGGGACATGTACAACC
CTAACCCAGCAGGACCAGGCATGGGGGGAGGACTGTTCTCAGACCTTTCCATGTCCACAATACCAAGG
TTTGATGATGGCACAACGTATATGGCAGATAGCTGTAGCTCAAACCCAGACCAACCAAGACAGCGGA
AGAAGCGCAAGCCATACACGCGCTACCAGACGATGGTCTTGGAGAACGAGTTCATGAGCAACAGTTA
CATCACGCGGCAGAAGAGATGGGAGATCAGTTGTAAGTTGCATCTGTCCGAGAGGCAAGTCAAAGTC
TGGTTCCAGAACCGCAGGATGAAGCGCAAGAAGCTGAACGAGAGAGCGAAGACTTTGATCAAGAGCG
ACTCAAGTAGCGATGGTCTGAGTACGACGCCCACCGGAAGCACAAACGGGACATTGATAACAAACGG
TGGCCTGAACGACCACCATCACCAGAAATAGCTGCCGCAGGGCCTACATGTGTGTCCTACGTCGCGTA
GTATAGATATGCATTGTAGAGATATGCGTAGCGGTGACAGTATCAGTGATTTCAACAGCAGCATTAAC
GGCACAATTAGTACCGGTACTTTTGGCAATGACAGCAGTATCAGCGATGGCGGCACTATCAGAAGCGA
CAGTGGCGTCAGCAGCTTAGGAAGTAGTGGTGGCAGCACACTCAAGTTGAACAGCTTCGCTAGCGCCC
CCGGTGGTTGTCACAGCGGTGCAGCAGCAGCAGCGGTGGCACATGACGTCACGCTAACTGAGGTCAT
GACCAATGACATCAGGAACAACATTCAGTCTCAACATGTTGGTTATTGCTGGTGACGTCACACATAGG
TACAAAATTATACTCAGCTGTCCATGTGACTTCTCTGTGTACTCAAGTGAGCCATACATGAACTAATTG
TTCAAATCAATTTGGGATTTGCTTACA

Figure S1. RNA samples from oocytes

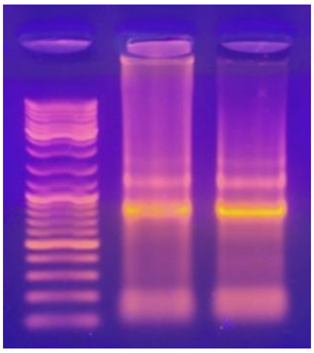

Figure S2. RT-RCR without Taq-Pol. Oocyte cDNA samples contain bands not caused by amplification

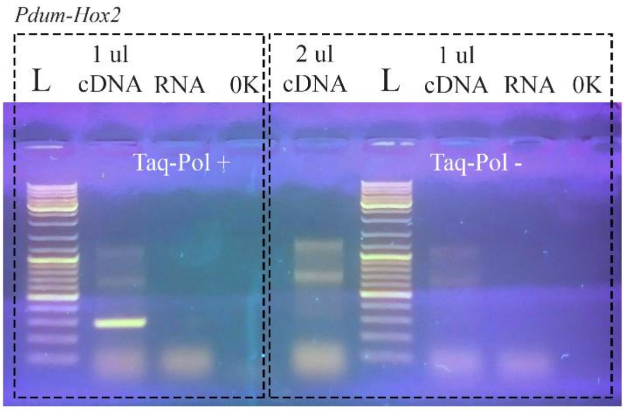

Figure S3. RT-PCR using intron-flanking primers

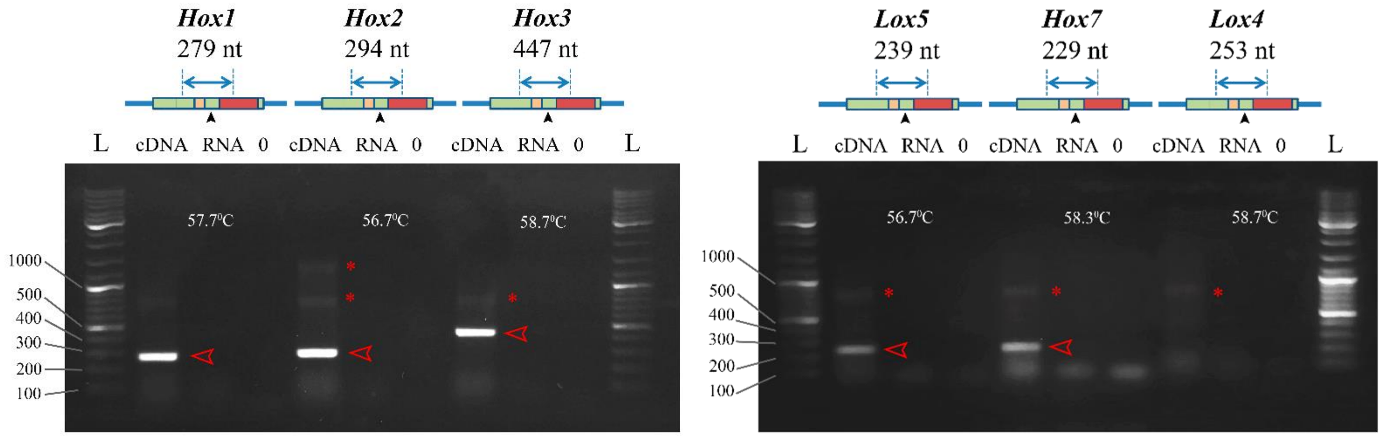

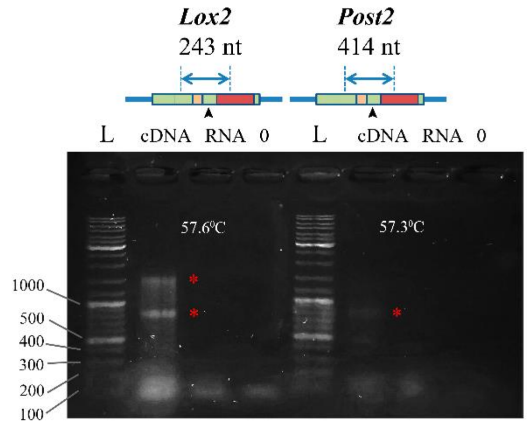

Schematic projection of primers onto Hox transcripts is shown at the top of each block . The expected size of the PCR-fragments is indicated above the double-headed arrow. Red rectangle - homeobox; yellow rectangle - hexapeptide sequence; coding region is marked in green; 5 '(left) and 3' (right) UTRs are marked in blue; the black arrowhead indicates the position of the intron. cDNA from oocytes was obtained using random primers. L-Ladder (GeneRuler DNA Ladder Mix); RNA - genomic DNA control; 0 - zero control, where water was used instead of the matrix. The white numbers indicate the annealling temperature. Bands of expected size marked with a hollow red arrowhead. Additional high bands (marked with red asterisks) visible on ~ 700-800 bp and above are not a product of amplification, since they are present in control samples that do not contain Taq-pol (**Figure S2**). These are residual rRNAs preserved in the oocyte cDNAs sample or the result of its reverse transcription.

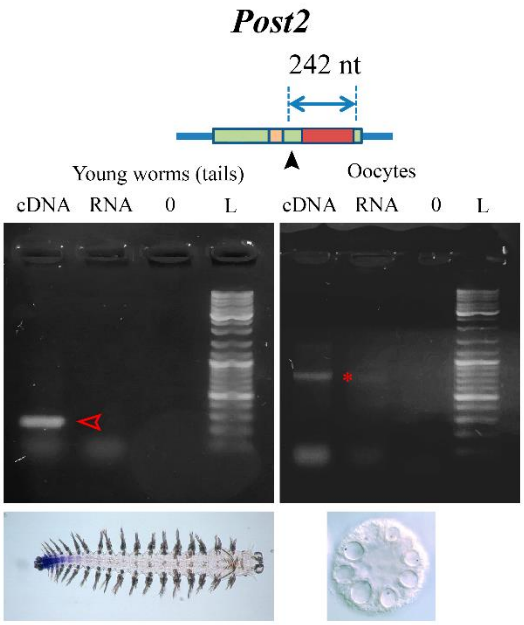

Figure S4. Results of check RT-PCR on cDNAs obtained from juvenile worm tails (left) and oocytes (right). Bottom shows in situ results with the Dig-RNA probe to *Pdum-Post2*. Nonspecific band (rRNAs) marked with red asterisks.

Figure S5. Strand-specific RT-PCR with two pairs of intron-flanking primers and sets of cDNAs obtained from juvenile worms and oocytes. The position of the primers is indicated at the top of the figure. It can be seen that only the outer pair of primers (F/R) works. We can also see that the efficiency of Strand-specific RT-PCR on cDNA matrices from tails is higher than on oocyte matrices. Random and Strand-specific (R) cDNAs from tails detected bands of the expected size (392 nt and 277 nt), indicating the presence of a sense transcript in the sample. In contrast, in samples from oocytes, the expected band is detected only in Random cDNA. R-Strand-specific cDNAs, show a low smear with no hint of the right size band. F-Strand-specific cDNAs show a very weak band at 392 nt and a brighter band at 300 nt, which cannot be considered as specific because it is absent from the Random sample.

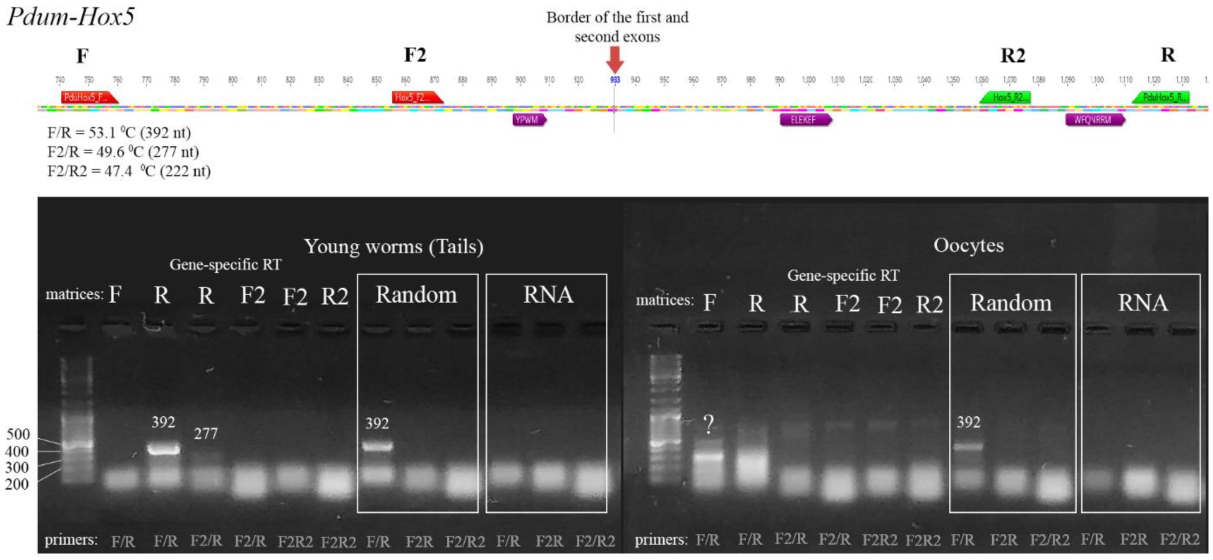

Figure S6. Endogenous alkaline phosphatase inhibition control (a) and Anti-Dig wash control (b)

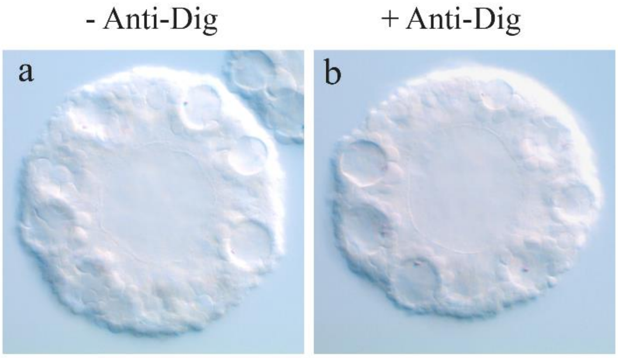

Figure S7. Results of in situ hybridization with antisense (a, b) and sense (c) Dig probes to *Pdum-Acox3*. (a, c) - equatorial slice; (b) - meridian slice. The star marks the pole, to which the transcript is shifted.

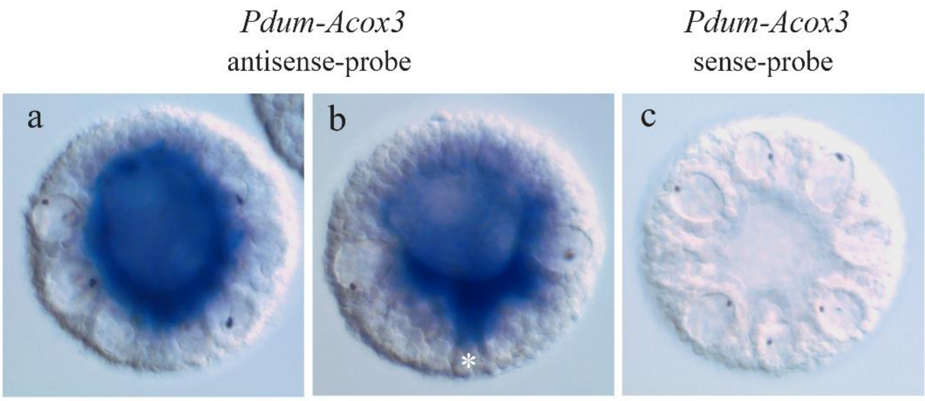

Figure S8. Example of a figure prepared to quantify the signal in the Fiji software

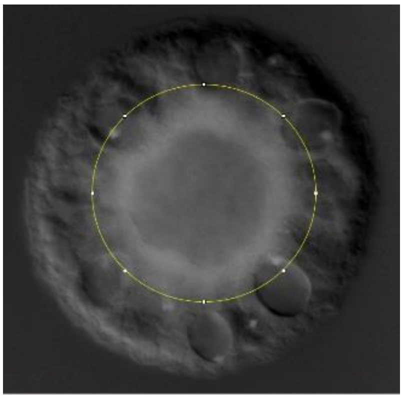

All images were centered, converted to 8-bit, and inverted. To assess the intensity of staining, we selected an area of the nucleus and a zone of cytoplasm around it, free of lipid droplets (Figure S8, yellow outline). Average, minimum and maximum staining intensities of the samples within the contour are obtained using the ROI manager's Measure command. The data are presented in Table S3. Further, the background mean values K1 (Anti-Dig +; Table S4) and K2 (*Acox3*as; Table S5) were subtracted from the mean values for each sample. The resulting numbers were used to build diagrams (Diagram S1 and Diagram S2).

Table S3. Signal intensity values for all samples

Genes	*Area*	*Mean*	*Min*	*Max*
K1 (Anti-Dig+)	260600	47.529	9	127
Acox3s	260600	185.577	80	223
K2 (Acox3as)	260600	46.931	18	137
Hox1s	260600	93.616	26	188
Hox1as	260600	77.153	11	121
Hox2s	260600	92.458	35	141
Hox3s	260600	53.191	6	100
Hox3as	260600	46.042	2	127
Hox4s	260600	79.805	28	130
Hox4as	260600	73.072	22	102
Hox5s	260600	90.725	23	141
Hox5as	260600	67.696	17	126
Lox5s	260600	80.138	20	158
Lox5as	260600	74.990	1	139
Hox7s	260600	96.937	21	153
Hox7as	260600	77.158	10	132
Lox4s	260600	79.949	25	124
Lox2s	260600	61.401	6	114
Lox2as	260600	69.616	9	126
Post2s	260600	58.031	0	149
Post2as	260600	55.880	0	137

Table S4. Signal intensity values after subtracting the antibodies' background

Genes	Mean (s)	Mean (as)	Subtraction K1 (s)	Subtraction K1(as)
K1 (Anti-Dig+)	47.529	47.529	0	0
Hox1	93.616	77.153	46,087	29,624
Hox3	53.191	46.042	5,622	-1,487
Hox4	79.805	73.072	32,276	25,543
Hox5	90.725	67.696	43,196	20,167
Lox5	80.138	74.990	32,609	27,461
Hox7	96.937	77.158	49,408	29,629
Lox2	61.401	69.616	13,872	22,087
Post2	58.031	55.880	10,502	8,351

Table S5. Signal intensity values after subtracting the *Pdum-Acox3* sense probe background

Genes	Mean (s)	Mean (as)	Subtraction K2 (s)	Subtraction K2 (as)
K2 (Acox3as)	46.931	46.931	0	0
Hox1	93.616	77.153	46,685	30.222
Hox3	53.191	46.042	6,26	-0,889
Hox4	79.805	73.072	32,874	26,141
Hox5	90.725	67.696	43,794	20,765
Lox5	80.138	74.990	33,207	28,059
Hox7	96.937	77.158	50,006	30,227
Lox2	61.401	69.616	14,47	22,685
Post2	58.031	55.880	11,1	8,949

Diagram S1. Diagram of the intensity of sense (s) and antisense (as) signals in situ after subtraction of the antibodies' background

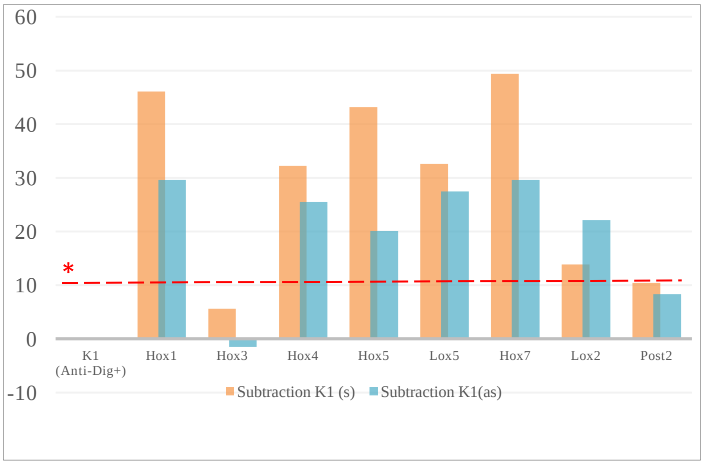

Diagram S2. Diagram of the intensity of sense (s) and antisense (as) signals in situ after subtracting of the *Pdum-Acox3* sense probe background

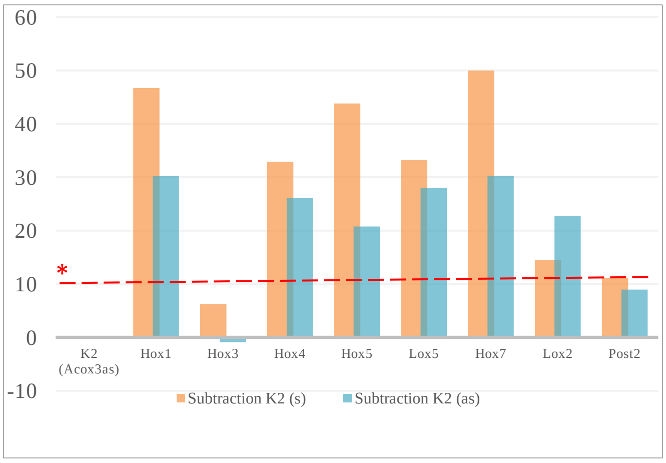

* The boundary of the background values, if we take the signal intensity Post2
